# Crystal structure of 8-[7,8-bis­(4-chloro­benzo­yl)-7*H*-cyclo­penta­[*a*]ace­naphthylen-9-yl]naphthalene-1-carb­oxy­lic acid

**DOI:** 10.1107/S2056989014026334

**Published:** 2015-01-01

**Authors:** Jomon P. Jacob, M. Sithambaresan, Christy Kunjachan, M. R. Prathapachandra Kurup

**Affiliations:** a Department of Applied Chemistry, Cochin University of Science and Technology, Kochi 682 022, India; b Department of Chemistry, Faculty of Science, Eastern University, Chenkalady, Sri Lanka

**Keywords:** crystal structure, domino reaction, acenaphthene­quinone, 4-chloro­aceto­phenone, hydrogen bonding

## Abstract

The title compound, C_40_H_22_Cl_2_O_4_, was formed by a Michael–Aldol domino reaction sequence, which coupled acenaphthene­quinone with 4-chloro­aceto­phenone in the presence of KOH in methanol. The dihedral angles between the central cyclo­penta­[*a*]ace­naphthyl­ene fused-ring system (r.m.s. deviation = 0.066 Å) and the 4-chloro­benzoyl rings are 62.25 (10) and 70.19 (10)°. The dihedral angle between the central ring system and the naphthoic acid grouping is 62.46 (7)°. This twisting of the pendant rings facilitates the formation of an intra­molecular aromatic π–π stacking inter­action between the 4-chloro­benzoyl and naphthoic acid rings, with centroid–centroid distances of 3.4533 (16) and 3.5311 (16) Å, and a C—H⋯π inter­action between one of the H atoms of the central moiety and the 4-chloro­benzoyl ring with an H⋯π distance of 2.57 Å. In the crystal, carb­oxy­lic acid inversion dimers generate *R*_2_^2^(8) loops. The dimers are linked by weak C—H⋯O and C—H⋯Cl hydrogen bonds and C—H⋯π inter­actions, generating a three-dimensional architecture.

## Abstract 2

The title compound packs with a three-dimensional supra­molecular architecture *via* O—H⋯O, C—H⋯O and C—H⋯Cl hydrogen bonds and through C—H⋯π inter­actions.

## Chemical context   

Domino reactions (Sousa *et al.*, 2014 ▸; Kumar & Perumal 2014 ▸; Pokhodylo *et al.*, 2014 ▸; Feng *et al.* 2014 ▸; Ramachandran *et al.*, 2014 ▸; Basetti *et al.*, 2014 ▸), also called cascade or tandem reactions, are usually carried out to enable the efficient construction of complex mol­ecules from simple substrates with high atom economy. In this reaction, multiple C—C or C—H bonds are formed in the same vessel, including different reaction mechanisms to form complex mol­ecules without the purification of inter­mediates. These reactions are often used in medical or combinatorial chemistry to synthesize complex active drug mol­ecules (Sudhapriya *et al.*, 2014 ▸; Tietze *et al.*, 2014 ▸; Fu *et al.*, 2013 ▸; Shestopalov *et al.*, 2013 ▸; Zohreh & Alizadeh, 2013 ▸; Renault *et al.*, 2007 ▸). Domino reactions are classified as homo-domino processes and hetero-domino processes (Nesi *et al.*, 1999 ▸).

**Figure 1:**
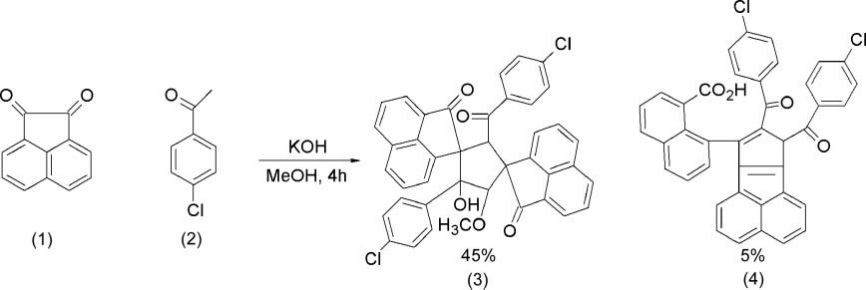
Reaction scheme showing the synthesis of the title compound (4).

One of the attractive strategies for constructing complex mol­ecules (Filippini *et al.*, 1995 ▸; List *et al.*, 2000 ▸; Wang *et al.*, 2007 ▸) is a domino sequence of Michael addition and aldol condensation. In this article, we report the formation of the title compound (4) through a domino reaction sequence involving Claisen–Schmidt condensation and benzil–benzilic acid rearrangement between acenaphthene­quinone (1) and 4-chloro­aceto­phenone (2) in the presence of methano­lic KOH (Fig. 1 ▸).
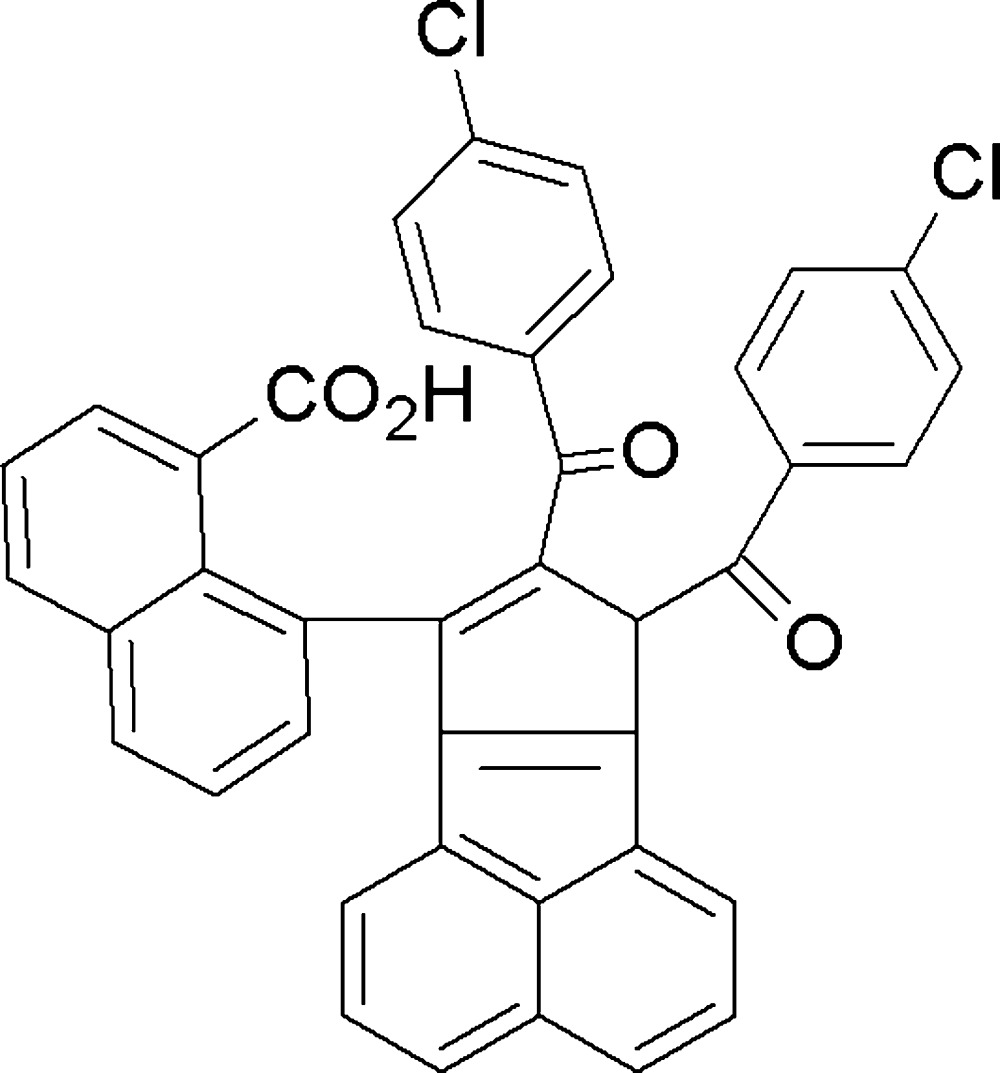


## Structural commentary   

**Figure 2:**
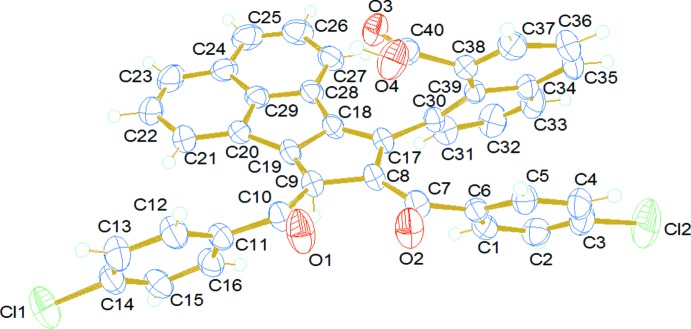
*ORTEP* view of the title compound, with atom labelling. Displacement ellipsoids are drawn at the 50% probability level.

In the title compound, the 4-chloro­benzoyl units are approximately coplanar with slight twisting [dihedral angle, 18.49 (13)°] and nearly parallel to the plane of naphthoic acid moiety with dihedral angles of 8.82 (11) and 12.06 (11)°. The C=O oxygen atoms of the two 4-chloro­benzoyl moieties point toward each other. The central cyclo­penta­[*a*]ace­naphthylene ring system makes dihedral angles of 62.25 (10) and 70.19 (10)° with the 4-chloro­benzoyl units and 62.46 (7)° with the naphthoic acid grouping. This twisting minimizes steric inter­actions among the substituents (Fig. 2 ▸) and facilitates the formation of intra­molecular π–π inter­actions between the 4-chloro­benzoyl and naphthoic acid rings with centroid centroid distances of 3.4533 (16) and 3.5311 (16) Å and a C—H⋯π inter­action between one of the hydrogen atoms of the central moiety and the 4-chloro­benzoyl ring.

## Supra­molecular features   

**Figure 3:**
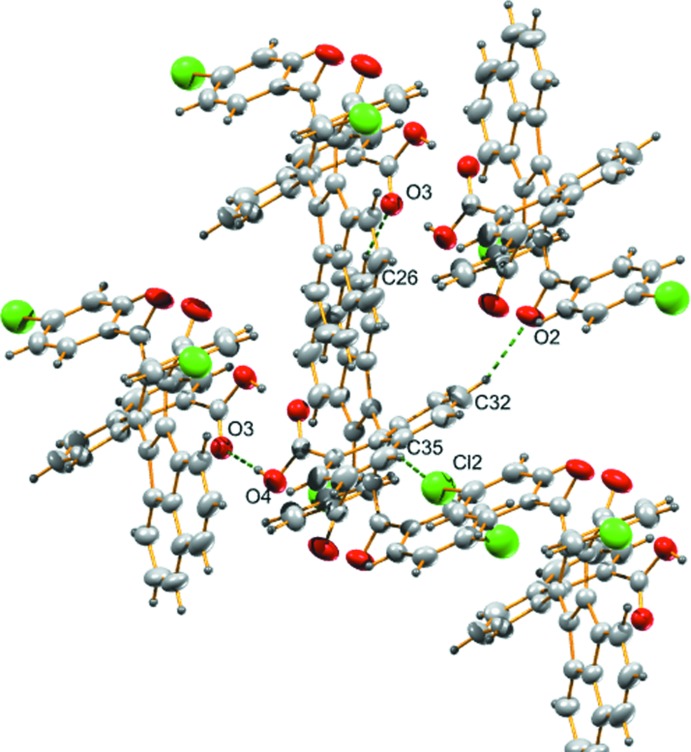
Hydrogen-bonding inter­actions (dashed lines) in the title compound.

**Figure 4:**
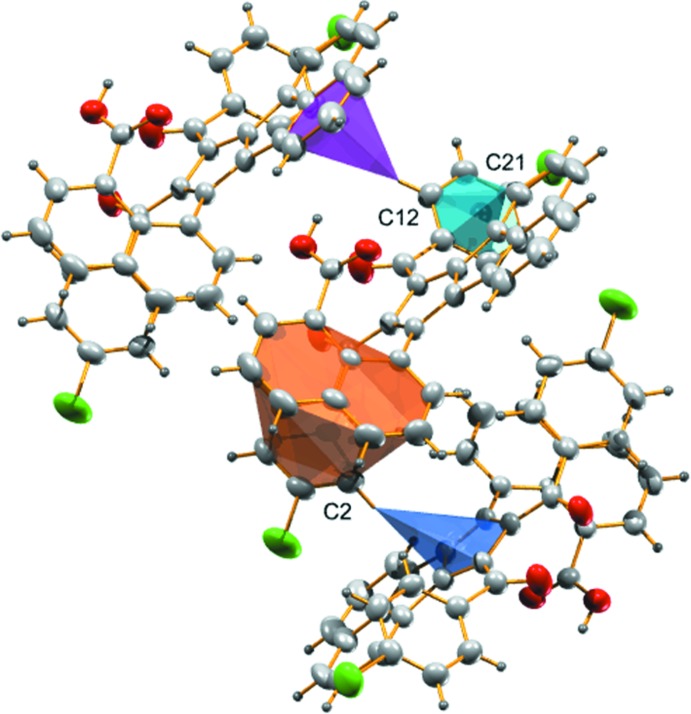
C—H⋯π and π–π inter­actions found in the title compound.

There are four inter­molecular hydrogen-bonding inter­actions present in the crystal. The carbonyl oxygen atoms (O2 and O3) accept three hydrogen bonds; one with the hydrogen atom from a carb­oxy­lic acid group of a neighboring mol­ecule with *D*⋯*A* distance of 2.649 (3) Å (−*x*, 1 − *y*, 2 − *z*) and the other two with the hydrogen atoms attached to atoms C32 and C26 of the naphthoic acid and cyclo­penta­[*a*]ace­naphthylene rings, respectively, of adjacent mol­ecules with *D*⋯*A* distances of 3.301 (4) (1 + *x*, *y*, *z*) and 3.416 (4) Å (1 − *x*, 1 − *y*, 2 − *z*) (Fig. 3 ▸). The fourth inter­action is between the H atom attached to the naphthoic acid ring and a chlorine atom of the 4-chloro­benzoyl moiety with a D⋯A distance of 3.619 (3) Å (1 − *x*, −*y*, 3 − *z*). Furthermore, there are two C—H⋯π inter­actions found between hydrogen atoms (H2 and H12) and the five- and six-membered rings of the cyclo­penta­[*a*]ace­naphthylene and 4-cholorobenzoyl moieties of neighbouring mol­ecules (Fig. 4 ▸), with H⋯π distances of 2.87 and 2.84 Å (Table 1 ▸).

**Figure 5:**
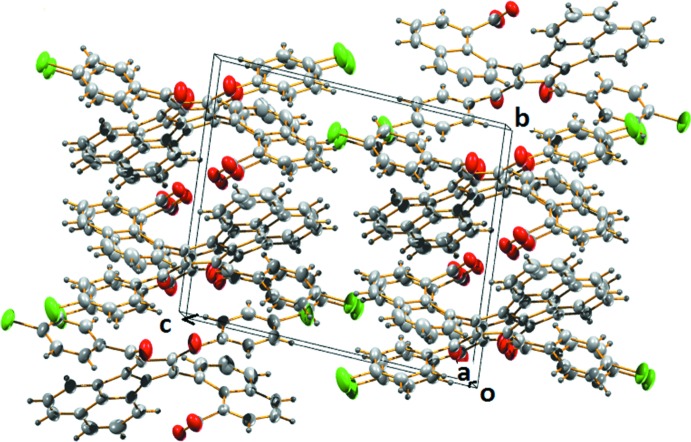
A packing diagram of the title compound viewed along the *a* axis.

The packing appears to be controlled by classical and non-classical hydrogen bonds and three C—H⋯π inter­actions (Mathew *et al.*, 2013 ▸). Fig. 5 ▸ shows the packing of the title compound viewed along the *a* axis.

## Synthesis and crystallization   

A mixture of acenaphthene­quinone (1) (4.6 g, 25 mmol), 4-chloro­aceto­phenone (2) (4.2 g, 27 mmol) and powdered potassium hydroxide (1.0 g) in methanol (30 ml) was stirred around 333 K for 4 h and later kept in a refrigerator for 48 h. The reaction mixture was concentrated and the residue was chromatographed over silica gel. Product (3) was obtained (Vadakkan *et al.*, 2003 ▸) by elution with a mixture (9:1) of hexane and ethyl acetate. Elution with a mixture of (1:1) methanol and ethyl acetate yielded the product (4) (Fig. 1 ▸). Red blocks of compound (4) were recrystallized from a solvent mixture of ethyl acetate and di­chloro­methane.

Yield 0.8 g (5%); m.p. >523 K; IR (KBr, ν_max_): 3370 (OH), 1732 (C=O) cm^−1^; ^1^H NMR (CDCl_3_): *δ* 8.00–5.30 (*m*, 20H, aromatic); ^13^C NMR (CDCl_3_): *δ* 207.57, 190.82, 179.39, 138.71, 135.57, 134.23, 134.17, 133.77, 132.57, 131.94, 131.69, 131.31, 130.40, 130.29, 129.90, 129.58, 129.22, 128.90, 128.85, 128.42, 128.06, 127.74, 127.66, 127.23, 126.54, 125.76, 125.64, 124.94, 124.38, 119.77, 103.38, 70.96; MS: *m*/*z* 636 (*M* ^+^); Analysis calculated for C_40_H_22_Cl_2_O_4_: C: 75.36, H: 3.48; found: C: 75.26, H: 3.30.

## Refinement   

Crystal data, data collection and structure refinement details are summarized in Table 2 ▸. All H atoms on C were placed in calculated positions, guided by difference maps, with C—H bond distances of 0.93 Å. H atoms were assigned as *U*_iso_(H) = 1.2U_eq_(C). Hydrogen atom H4′ of the naphthoic acid group was located from a difference Fourier map and refined with a distance restraint of O—H = 0.84 (1) Å. The low-angle reflections (001), (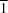
01) and (0
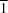
1) were omitted from the refinement owing to bad agreement.

## Supplementary Material

Crystal structure: contains datablock(s) I. DOI: 10.1107/S2056989014026334/hb7324sup1.cif

Structure factors: contains datablock(s) I. DOI: 10.1107/S2056989014026334/hb7324Isup2.hkl

CCDC reference: 1024474

Additional supporting information:  crystallographic information; 3D view; checkCIF report

## Figures and Tables

**Table 1:** Hydrogen-bond geometry (, ) *Cg*1 is the centroid of the C18C20/C28/C29 ring, *Cg*2 is the centroid of the C24C29 ring and *Cg*3 is the centroid of the C11C16 ring.

*D*H*A*	*D*H	H*A*	*D* *A*	*D*H*A*
O4H4O3^i^	0.84(1)	1.81(1)	2.649(3)	178(4)
C26H26O3^ii^	0.93	2.52	3.416(4)	163
C32H32O2^iii^	0.93	2.47	3.301(4)	149
C35H35Cl2^iv^	0.93	2.74	3.619(3)	157
C2H2*Cg*1^v^	0.93	2.87	3.577(3)	134
C12H12*Cg*2^vi^	0.93	2.84	3.725(3)	160
C21H21*Cg*3	0.93	2.57	3.425(3)	152

Symmetry codes: (i) 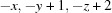; (ii) 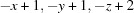; (iii) 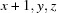; (iv) 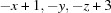; (v) 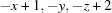; (vi) 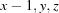.

**Table 2:** Experimental details

Crystal data	
Chemical formula	C_40_H_22_Cl_2_O_4_
*M*_r_	637.47
Crystal system, space group	Triclinic, *P* 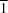
Temperature (K)	296
*a*, *b*, *c* ()	9.1617(6), 12.5518(8), 13.9305(8)
, , ()	84.669(3), 88.468(3), 72.364(3)
*V* (^3^)	1520.05(17)
*Z*	2
Radiation type	Mo *K*
(mm^1^)	0.26
Crystal size (mm)	0.35 0.30 0.25

Data collection	
Diffractometer	Bruker Kappa APEXII CCD
Absorption correction	Multi-scan (*SADABS*; Bruker, 2004 ▸)
*T*_min_, *T*_max_	0.891, 0.908
No. of measured, independent and observed [*I* > 2(*I*)] reflections	19996, 5287, 4251
*R*_int_	0.033
(sin /)_max_ (^1^)	0.595

Refinement	
*R*[*F* ^2^ > 2(*F* ^2^)], *wR*(*F* ^2^), *S*	0.052, 0.152, 1.12
No. of reflections	5287
No. of parameters	419
No. of restraints	1
H-atom treatment	H atoms treated by a mixture of independent and constrained refinement
_max_, _min_ (e ^3^)	0.51, 0.78

Computer programs:*APEX2*, *SAINT* and *XPREP* (Bruker, 2004 ▸), *SHELXS97*, *SHELXL97* and *SHELXL2014* (Sheldrick, 2008 ▸), *ORTEP-3 for Windows* (Farrugia, 2012 ▸), *DIAMOND* (Brandenburg, 2010 ▸), and *publCIF* (Westrip, 2010 ▸).

## supplementary crystallographic information

### Crystal data

**Table:**

C_40_H_22_Cl_2_O_4_	*Z* = 2
*M**_r_* = 637.47	*F*(000) = 656
Triclinic, *P*1	*D*_x_ = 1.393 Mg m^−^^3^
*a* = 9.1617 (6) Å	Mo *K*α radiation, λ = 0.71073 Å
*b* = 12.5518 (8) Å	Cell parameters from 9963 reflections
*c* = 13.9305 (8) Å	θ = 2.4–28.1°
α = 84.669 (3)°	µ = 0.26 mm^−^^1^
β = 88.468 (3)°	*T* = 296 K
γ = 72.364 (3)°	Block, red
*V* = 1520.05 (17) Å^3^	0.35 × 0.30 × 0.25 mm

### Data collection

**Table:**

Bruker axs kappa apex2 CCD Diffractometer	4251 reflections with *I* > 2σ(*I*)
ω and φ scan	*R*_int_ = 0.033
Absorption correction: multi-scan (*SADABS*; Bruker, 2004)	θ_max_ = 25.0°, θ_min_ = 2.2°
*T*_min_ = 0.891, *T*_max_ = 0.908	*h* = −10→10
19996 measured reflections	*k* = −14→14
5287 independent reflections	*l* = −16→16

### Refinement

**Table:**

Refinement on *F*^2^	1 restraint
Least-squares matrix: full	Hydrogen site location: mixed
*R*[*F*^2^ > 2σ(*F*^2^)] = 0.055	H atoms treated by a mixture of independent and constrained refinement
*wR*(*F*^2^) = 0.154	*w* = 1/[σ^2^(*F*_o_^2^) + (0.0529*P*)^2^ + 1.3725*P*] where *P* = (*F*_o_^2^ + 2*F*_c_^2^)/3
*S* = 1.16	(Δ/σ)_max_ < 0.001
5287 reflections	Δρ_max_ = 0.51 e Å^−^^3^
419 parameters	Δρ_min_ = −0.78 e Å^−^^3^

### Special details

**Table:**

Geometry. All esds (except the esd in the dihedral angle between two l.s. planes) are estimated using the full covariance matrix. The cell esds are taken into account individually in the estimation of esds in distances, angles and torsion angles; correlations between esds in cell parameters are only used when they are defined by crystal symmetry. An approximate (isotropic) treatment of cell esds is used for estimating esds involving l.s. planes.

### Fractional atomic coordinates and isotropic or equivalent isotropic displacement parameters (Å^2^)

**Table:**

	*x*	*y*	*z*	*U*_iso_*/*U*_eq_	
C1	0.3710 (3)	−0.0055 (2)	1.14118 (19)	0.0423 (6)	
H1	0.4150	−0.0104	1.0802	0.051*	
C2	0.4574 (3)	−0.0622 (2)	1.2201 (2)	0.0494 (7)	
H2	0.5584	−0.1061	1.2129	0.059*	
C3	0.3904 (4)	−0.0521 (3)	1.3095 (2)	0.0534 (7)	
C4	0.2397 (4)	0.0087 (2)	1.32183 (19)	0.0497 (7)	
H4	0.1965	0.0132	1.3831	0.060*	
C5	0.1536 (3)	0.0630 (2)	1.24211 (18)	0.0410 (6)	
H5	0.0508	0.1027	1.2492	0.049*	
C6	0.2204 (3)	0.0584 (2)	1.15107 (17)	0.0346 (5)	
C7	0.1283 (3)	0.1184 (2)	1.06661 (18)	0.0389 (6)	
C8	0.1805 (3)	0.1835 (2)	0.99406 (16)	0.0354 (5)	
C9	0.1165 (3)	0.2180 (2)	0.89523 (16)	0.0355 (5)	
H9	0.1824	0.1478	0.8726	0.043*	
C10	−0.0153 (3)	0.1981 (2)	0.85791 (18)	0.0429 (6)	
C11	−0.0373 (3)	0.2001 (2)	0.75185 (18)	0.0395 (6)	
C12	−0.1801 (3)	0.2556 (2)	0.7114 (2)	0.0491 (7)	
H12	−0.2595	0.2938	0.7501	0.059*	
C13	−0.2052 (3)	0.2546 (3)	0.6143 (2)	0.0525 (7)	
H13	−0.2998	0.2939	0.5867	0.063*	
C14	−0.0878 (3)	0.1946 (2)	0.55900 (19)	0.0476 (7)	
C15	0.0539 (3)	0.1368 (3)	0.5976 (2)	0.0494 (7)	
H15	0.1315	0.0958	0.5592	0.059*	
C16	0.0788 (3)	0.1407 (2)	0.6946 (2)	0.0458 (6)	
H16	0.1745	0.1031	0.7215	0.055*	
C17	0.3071 (3)	0.2282 (2)	1.00154 (16)	0.0356 (5)	
C18	0.3212 (3)	0.2850 (2)	0.91319 (17)	0.0370 (6)	
C19	0.2079 (3)	0.2776 (2)	0.84755 (16)	0.0369 (6)	
C20	0.2216 (3)	0.3468 (2)	0.75736 (17)	0.0395 (6)	
C21	0.1484 (4)	0.3781 (2)	0.66922 (19)	0.0513 (7)	
H21	0.0674	0.3521	0.6547	0.062*	
C22	0.1988 (4)	0.4506 (3)	0.6011 (2)	0.0585 (8)	
H22	0.1514	0.4695	0.5411	0.070*	
C23	0.3133 (4)	0.4933 (3)	0.6199 (2)	0.0620 (9)	
H23	0.3423	0.5405	0.5729	0.074*	
C24	0.3892 (4)	0.4669 (2)	0.7103 (2)	0.0500 (7)	
C25	0.5021 (4)	0.5104 (3)	0.7430 (3)	0.0631 (9)	
H25	0.5372	0.5603	0.7022	0.076*	
C26	0.5604 (4)	0.4798 (3)	0.8343 (3)	0.0616 (8)	
H26	0.6338	0.5104	0.8544	0.074*	
C27	0.5130 (3)	0.4036 (2)	0.8989 (2)	0.0491 (7)	
H27	0.5551	0.3837	0.9604	0.059*	
C28	0.4040 (3)	0.3594 (2)	0.86951 (17)	0.0390 (6)	
C29	0.3416 (3)	0.3925 (2)	0.77553 (18)	0.0410 (6)	
C30	0.4027 (3)	0.2227 (2)	1.08743 (17)	0.0359 (5)	
C31	0.5582 (3)	0.1748 (3)	1.0823 (2)	0.0483 (7)	
H31	0.6034	0.1592	1.0226	0.058*	
C32	0.6509 (3)	0.1488 (3)	1.1650 (2)	0.0596 (8)	
H32	0.7566	0.1189	1.1595	0.072*	
C33	0.5861 (3)	0.1672 (3)	1.2529 (2)	0.0560 (8)	
H33	0.6466	0.1433	1.3080	0.067*	
C34	0.4282 (3)	0.2221 (2)	1.26200 (18)	0.0424 (6)	
C35	0.3591 (4)	0.2439 (3)	1.35317 (19)	0.0553 (8)	
H35	0.4190	0.2199	1.4085	0.066*	
C36	0.2086 (4)	0.2987 (3)	1.3618 (2)	0.0601 (9)	
H36	0.1642	0.3073	1.4225	0.072*	
C37	0.1190 (4)	0.3425 (2)	1.2789 (2)	0.0529 (7)	
H37	0.0163	0.3830	1.2850	0.063*	
C38	0.1818 (3)	0.3261 (2)	1.18879 (17)	0.0376 (6)	
C39	0.3359 (3)	0.2582 (2)	1.17763 (16)	0.0337 (5)	
C40	0.0952 (3)	0.3994 (2)	1.10642 (19)	0.0402 (6)	
O1	−0.1211 (2)	0.1783 (2)	0.90948 (15)	0.0642 (6)	
O2	−0.0068 (2)	0.1055 (2)	1.06624 (14)	0.0571 (6)	
O3	0.1576 (2)	0.45386 (16)	1.05156 (14)	0.0489 (5)	
O4	−0.0494 (2)	0.40610 (19)	1.10215 (16)	0.0585 (6)	
Cl1	−0.12238 (12)	0.18916 (9)	0.43778 (6)	0.0775 (3)	
Cl2	0.50109 (14)	−0.11781 (11)	1.40953 (8)	0.1010 (4)	
H4'	−0.082 (5)	0.451 (3)	1.053 (2)	0.100 (15)*	

### Atomic displacement parameters (Å^2^)

**Table:**

	*U*^11^	*U*^22^	*U*^33^	*U*^12^	*U*^13^	*U*^23^
C1	0.0453 (15)	0.0426 (14)	0.0383 (14)	−0.0109 (12)	0.0040 (11)	−0.0090 (11)
C2	0.0440 (16)	0.0444 (15)	0.0557 (17)	−0.0074 (13)	−0.0055 (13)	−0.0022 (13)
C3	0.062 (2)	0.0541 (17)	0.0449 (16)	−0.0218 (15)	−0.0142 (14)	0.0106 (13)
C4	0.0630 (19)	0.0581 (17)	0.0326 (14)	−0.0273 (15)	0.0041 (13)	0.0017 (12)
C5	0.0403 (14)	0.0481 (15)	0.0349 (13)	−0.0148 (12)	0.0042 (11)	−0.0010 (11)
C6	0.0394 (14)	0.0367 (13)	0.0318 (12)	−0.0176 (11)	0.0012 (10)	−0.0030 (10)
C7	0.0382 (14)	0.0475 (15)	0.0328 (13)	−0.0149 (12)	0.0010 (10)	−0.0056 (11)
C8	0.0358 (13)	0.0457 (14)	0.0264 (11)	−0.0139 (11)	−0.0019 (10)	−0.0060 (10)
C9	0.0369 (13)	0.0408 (13)	0.0289 (12)	−0.0116 (11)	−0.0029 (10)	−0.0040 (10)
C10	0.0442 (15)	0.0511 (15)	0.0349 (13)	−0.0162 (13)	−0.0055 (11)	−0.0041 (11)
C11	0.0399 (14)	0.0447 (14)	0.0375 (13)	−0.0169 (12)	−0.0076 (11)	−0.0053 (11)
C12	0.0461 (16)	0.0570 (17)	0.0407 (15)	−0.0079 (13)	−0.0055 (12)	−0.0112 (13)
C13	0.0477 (17)	0.0632 (18)	0.0433 (15)	−0.0114 (14)	−0.0118 (13)	−0.0026 (13)
C14	0.0561 (18)	0.0574 (17)	0.0345 (14)	−0.0233 (14)	−0.0061 (12)	−0.0076 (12)
C15	0.0497 (17)	0.0569 (17)	0.0436 (15)	−0.0156 (14)	0.0018 (13)	−0.0169 (13)
C16	0.0395 (15)	0.0504 (16)	0.0471 (15)	−0.0114 (13)	−0.0096 (12)	−0.0063 (12)
C17	0.0381 (14)	0.0432 (13)	0.0262 (11)	−0.0121 (11)	−0.0011 (10)	−0.0062 (10)
C18	0.0424 (14)	0.0433 (14)	0.0274 (12)	−0.0146 (11)	0.0014 (10)	−0.0081 (10)
C19	0.0452 (15)	0.0424 (14)	0.0249 (11)	−0.0146 (12)	−0.0007 (10)	−0.0065 (10)
C20	0.0532 (16)	0.0360 (13)	0.0300 (12)	−0.0139 (12)	0.0002 (11)	−0.0056 (10)
C21	0.071 (2)	0.0480 (16)	0.0335 (14)	−0.0162 (15)	−0.0075 (13)	−0.0021 (12)
C22	0.087 (2)	0.0489 (17)	0.0355 (15)	−0.0166 (17)	−0.0058 (15)	0.0035 (12)
C23	0.093 (3)	0.0475 (17)	0.0427 (16)	−0.0210 (17)	0.0091 (16)	0.0076 (13)
C24	0.069 (2)	0.0363 (14)	0.0459 (16)	−0.0185 (14)	0.0091 (14)	−0.0044 (12)
C25	0.080 (2)	0.0496 (17)	0.068 (2)	−0.0344 (17)	0.0146 (18)	−0.0007 (15)
C26	0.069 (2)	0.0560 (18)	0.073 (2)	−0.0374 (17)	0.0065 (17)	−0.0108 (16)
C27	0.0566 (18)	0.0527 (16)	0.0451 (15)	−0.0253 (14)	0.0013 (13)	−0.0109 (13)
C28	0.0458 (15)	0.0416 (14)	0.0324 (13)	−0.0164 (12)	0.0048 (11)	−0.0083 (10)
C29	0.0531 (16)	0.0366 (13)	0.0353 (13)	−0.0157 (12)	0.0069 (11)	−0.0074 (10)
C30	0.0350 (13)	0.0437 (14)	0.0303 (12)	−0.0138 (11)	−0.0048 (10)	−0.0015 (10)
C31	0.0374 (15)	0.0632 (18)	0.0455 (15)	−0.0170 (13)	0.0016 (12)	−0.0058 (13)
C32	0.0335 (15)	0.079 (2)	0.065 (2)	−0.0185 (15)	−0.0123 (14)	0.0072 (17)
C33	0.0492 (18)	0.069 (2)	0.0524 (18)	−0.0242 (15)	−0.0258 (14)	0.0110 (15)
C34	0.0545 (17)	0.0419 (14)	0.0343 (13)	−0.0204 (13)	−0.0119 (12)	0.0024 (11)
C35	0.085 (2)	0.0581 (18)	0.0294 (14)	−0.0312 (18)	−0.0151 (14)	0.0025 (12)
C36	0.093 (3)	0.0601 (19)	0.0286 (14)	−0.0247 (19)	0.0057 (15)	−0.0093 (13)
C37	0.067 (2)	0.0442 (15)	0.0418 (15)	−0.0078 (14)	0.0091 (14)	−0.0083 (12)
C38	0.0475 (15)	0.0338 (13)	0.0317 (12)	−0.0121 (11)	−0.0002 (11)	−0.0038 (10)
C39	0.0401 (14)	0.0356 (12)	0.0281 (12)	−0.0157 (11)	−0.0053 (10)	−0.0005 (9)
C40	0.0396 (15)	0.0392 (14)	0.0385 (14)	−0.0064 (11)	−0.0022 (11)	−0.0046 (11)
O1	0.0481 (12)	0.1076 (19)	0.0438 (11)	−0.0367 (13)	−0.0076 (9)	0.0055 (11)
O2	0.0446 (12)	0.0895 (16)	0.0437 (11)	−0.0339 (11)	−0.0035 (9)	0.0091 (10)
O3	0.0452 (11)	0.0517 (11)	0.0462 (11)	−0.0126 (9)	−0.0090 (9)	0.0099 (9)
O4	0.0407 (12)	0.0666 (14)	0.0605 (14)	−0.0107 (10)	−0.0047 (10)	0.0153 (11)
Cl1	0.0868 (7)	0.1123 (8)	0.0380 (4)	−0.0333 (6)	−0.0096 (4)	−0.0165 (4)
Cl2	0.0940 (8)	0.1317 (10)	0.0678 (6)	−0.0311 (7)	−0.0373 (6)	0.0394 (6)

### Geometric parameters (Å, º)

**Table:**

C1—C2	1.379 (4)	C20—C29	1.423 (4)
C1—C6	1.380 (4)	C21—C22	1.418 (4)
C1—H1	0.9300	C21—H21	0.9300
C2—C3	1.373 (4)	C22—C23	1.355 (5)
C2—H2	0.9300	C22—H22	0.9300
C3—C4	1.376 (4)	C23—C24	1.418 (4)
C3—Cl2	1.734 (3)	C23—H23	0.9300
C4—C5	1.378 (4)	C24—C29	1.398 (4)
C4—H4	0.9300	C24—C25	1.411 (5)
C5—C6	1.392 (3)	C25—C26	1.368 (5)
C5—H5	0.9300	C25—H25	0.9300
C6—C7	1.472 (3)	C26—C27	1.408 (4)
C7—O2	1.297 (3)	C26—H26	0.9300
C7—C8	1.405 (3)	C27—C28	1.369 (4)
C8—C17	1.443 (3)	C27—H27	0.9300
C8—C9	1.485 (3)	C28—C29	1.417 (4)
C9—C19	1.399 (3)	C30—C31	1.372 (4)
C9—C10	1.425 (4)	C30—C39	1.431 (3)
C9—H9	0.9800	C31—C32	1.403 (4)
C10—O1	1.262 (3)	C31—H31	0.9300
C10—C11	1.493 (3)	C32—C33	1.355 (5)
C11—C16	1.381 (4)	C32—H32	0.9300
C11—C12	1.389 (4)	C33—C34	1.409 (4)
C12—C13	1.381 (4)	C33—H33	0.9300
C12—H12	0.9300	C34—C35	1.416 (4)
C13—C14	1.375 (4)	C34—C39	1.423 (3)
C13—H13	0.9300	C35—C36	1.350 (5)
C14—C15	1.377 (4)	C35—H35	0.9300
C14—Cl1	1.738 (3)	C36—C37	1.403 (4)
C15—C16	1.385 (4)	C36—H36	0.9300
C15—H15	0.9300	C37—C38	1.373 (4)
C16—H16	0.9300	C37—H37	0.9300
C17—C18	1.387 (3)	C38—C39	1.424 (4)
C17—C30	1.486 (3)	C38—C40	1.484 (4)
C18—C19	1.431 (3)	C40—O3	1.222 (3)
C18—C28	1.455 (4)	C40—O4	1.305 (3)
C19—C20	1.485 (3)	O4—H4'	0.842 (10)
C20—C21	1.381 (4)		
			
C2—C1—C6	121.2 (3)	C29—C20—C19	105.0 (2)
C2—C1—H1	119.4	C20—C21—C22	118.9 (3)
C6—C1—H1	119.4	C20—C21—H21	120.6
C3—C2—C1	118.2 (3)	C22—C21—H21	120.6
C3—C2—H2	120.9	C23—C22—C21	122.8 (3)
C1—C2—H2	120.9	C23—C22—H22	118.6
C2—C3—C4	122.1 (3)	C21—C22—H22	118.6
C2—C3—Cl2	118.4 (3)	C22—C23—C24	120.7 (3)
C4—C3—Cl2	119.4 (2)	C22—C23—H23	119.6
C3—C4—C5	119.0 (3)	C24—C23—H23	119.6
C3—C4—H4	120.5	C29—C24—C25	116.7 (3)
C5—C4—H4	120.5	C29—C24—C23	115.8 (3)
C4—C5—C6	120.1 (3)	C25—C24—C23	127.5 (3)
C4—C5—H5	120.0	C26—C25—C24	120.5 (3)
C6—C5—H5	120.0	C26—C25—H25	119.7
C1—C6—C5	119.3 (2)	C24—C25—H25	119.7
C1—C6—C7	121.1 (2)	C25—C26—C27	122.3 (3)
C5—C6—C7	119.6 (2)	C25—C26—H26	118.9
O2—C7—C8	123.3 (2)	C27—C26—H26	118.9
O2—C7—C6	112.9 (2)	C28—C27—C26	118.8 (3)
C8—C7—C6	123.7 (2)	C28—C27—H27	120.6
C7—C8—C17	126.3 (2)	C26—C27—H27	120.6
C7—C8—C9	126.3 (2)	C27—C28—C29	119.0 (2)
C17—C8—C9	107.4 (2)	C27—C28—C18	136.0 (2)
C19—C9—C10	127.0 (2)	C29—C28—C18	104.9 (2)
C19—C9—C8	106.3 (2)	C24—C29—C28	122.7 (3)
C10—C9—C8	126.7 (2)	C24—C29—C20	124.3 (3)
C19—C9—H9	90.7	C28—C29—C20	112.9 (2)
C10—C9—H9	90.7	C31—C30—C39	118.9 (2)
C8—C9—H9	90.7	C31—C30—C17	119.2 (2)
O1—C10—C9	124.1 (2)	C39—C30—C17	121.7 (2)
O1—C10—C11	114.9 (2)	C30—C31—C32	121.6 (3)
C9—C10—C11	121.0 (2)	C30—C31—H31	119.2
C16—C11—C12	119.5 (2)	C32—C31—H31	119.2
C16—C11—C10	121.0 (2)	C33—C32—C31	119.8 (3)
C12—C11—C10	119.4 (2)	C33—C32—H32	120.1
C13—C12—C11	120.5 (3)	C31—C32—H32	120.1
C13—C12—H12	119.7	C32—C33—C34	120.9 (3)
C11—C12—H12	119.7	C32—C33—H33	119.6
C14—C13—C12	118.8 (3)	C34—C33—H33	119.6
C14—C13—H13	120.6	C33—C34—C35	121.6 (3)
C12—C13—H13	120.6	C33—C34—C39	119.4 (3)
C13—C14—C15	121.9 (3)	C35—C34—C39	119.0 (3)
C13—C14—Cl1	118.6 (2)	C36—C35—C34	121.6 (3)
C15—C14—Cl1	119.5 (2)	C36—C35—H35	119.2
C14—C15—C16	118.8 (3)	C34—C35—H35	119.2
C14—C15—H15	120.6	C35—C36—C37	119.8 (3)
C16—C15—H15	120.6	C35—C36—H36	120.1
C11—C16—C15	120.5 (3)	C37—C36—H36	120.1
C11—C16—H16	119.7	C38—C37—C36	120.6 (3)
C15—C16—H16	119.7	C38—C37—H37	119.7
C18—C17—C8	107.4 (2)	C36—C37—H37	119.7
C18—C17—C30	124.2 (2)	C37—C38—C39	120.7 (2)
C8—C17—C30	128.3 (2)	C37—C38—C40	117.1 (2)
C17—C18—C19	110.0 (2)	C39—C38—C40	121.0 (2)
C17—C18—C28	139.7 (2)	C34—C39—C38	117.6 (2)
C19—C18—C28	109.9 (2)	C34—C39—C30	118.2 (2)
C9—C19—C18	108.9 (2)	C38—C39—C30	124.2 (2)
C9—C19—C20	143.3 (2)	O3—C40—O4	124.3 (2)
C18—C19—C20	107.2 (2)	O3—C40—C38	120.0 (2)
C21—C20—C29	117.4 (2)	O4—C40—C38	115.5 (2)
C21—C20—C19	137.5 (3)	C40—O4—H4'	104 (3)
			
C6—C1—C2—C3	−1.0 (4)	C29—C20—C21—C22	1.8 (4)
C1—C2—C3—C4	2.5 (5)	C19—C20—C21—C22	178.7 (3)
C1—C2—C3—Cl2	−177.0 (2)	C20—C21—C22—C23	−2.0 (5)
C2—C3—C4—C5	−1.1 (5)	C21—C22—C23—C24	0.0 (5)
Cl2—C3—C4—C5	178.4 (2)	C22—C23—C24—C29	2.0 (5)
C3—C4—C5—C6	−1.8 (4)	C22—C23—C24—C25	−175.2 (3)
C2—C1—C6—C5	−1.8 (4)	C29—C24—C25—C26	0.1 (5)
C2—C1—C6—C7	−179.7 (2)	C23—C24—C25—C26	177.3 (3)
C4—C5—C6—C1	3.2 (4)	C24—C25—C26—C27	0.8 (5)
C4—C5—C6—C7	−178.8 (2)	C25—C26—C27—C28	−0.5 (5)
C1—C6—C7—O2	133.8 (3)	C26—C27—C28—C29	−0.6 (4)
C5—C6—C7—O2	−44.1 (3)	C26—C27—C28—C18	−176.6 (3)
C1—C6—C7—C8	−46.5 (4)	C17—C18—C28—C27	3.0 (6)
C5—C6—C7—C8	135.6 (3)	C19—C18—C28—C27	174.3 (3)
O2—C7—C8—C17	161.1 (3)	C17—C18—C28—C29	−173.4 (3)
C6—C7—C8—C17	−18.6 (4)	C19—C18—C28—C29	−2.2 (3)
O2—C7—C8—C9	−19.6 (4)	C25—C24—C29—C28	−1.2 (4)
C6—C7—C8—C9	160.8 (2)	C23—C24—C29—C28	−178.8 (3)
C7—C8—C9—C19	−177.7 (2)	C25—C24—C29—C20	175.4 (3)
C17—C8—C9—C19	1.8 (3)	C23—C24—C29—C20	−2.2 (4)
C7—C8—C9—C10	4.5 (4)	C27—C28—C29—C24	1.5 (4)
C17—C8—C9—C10	−176.0 (3)	C18—C28—C29—C24	178.7 (2)
C19—C9—C10—O1	−155.4 (3)	C27—C28—C29—C20	−175.5 (2)
C8—C9—C10—O1	21.9 (5)	C18—C28—C29—C20	1.7 (3)
C19—C9—C10—C11	24.2 (4)	C21—C20—C29—C24	0.3 (4)
C8—C9—C10—C11	−158.5 (2)	C19—C20—C29—C24	−177.5 (2)
O1—C10—C11—C16	−131.2 (3)	C21—C20—C29—C28	177.2 (2)
C9—C10—C11—C16	49.2 (4)	C19—C20—C29—C28	−0.6 (3)
O1—C10—C11—C12	44.0 (4)	C18—C17—C30—C31	−61.0 (4)
C9—C10—C11—C12	−135.7 (3)	C8—C17—C30—C31	122.7 (3)
C16—C11—C12—C13	−2.0 (4)	C18—C17—C30—C39	123.9 (3)
C10—C11—C12—C13	−177.2 (3)	C8—C17—C30—C39	−52.3 (4)
C11—C12—C13—C14	2.2 (5)	C39—C30—C31—C32	7.0 (4)
C12—C13—C14—C15	−0.7 (5)	C17—C30—C31—C32	−168.2 (3)
C12—C13—C14—Cl1	177.5 (2)	C30—C31—C32—C33	2.4 (5)
C13—C14—C15—C16	−0.9 (5)	C31—C32—C33—C34	−6.1 (5)
Cl1—C14—C15—C16	−179.1 (2)	C32—C33—C34—C35	−178.8 (3)
C12—C11—C16—C15	0.3 (4)	C32—C33—C34—C39	0.3 (4)
C10—C11—C16—C15	175.5 (3)	C33—C34—C35—C36	178.6 (3)
C14—C15—C16—C11	1.1 (4)	C39—C34—C35—C36	−0.6 (4)
C7—C8—C17—C18	178.7 (2)	C34—C35—C36—C37	−4.6 (5)
C9—C8—C17—C18	−0.7 (3)	C35—C36—C37—C38	2.8 (5)
C7—C8—C17—C30	−4.5 (4)	C36—C37—C38—C39	4.2 (4)
C9—C8—C17—C30	176.1 (2)	C36—C37—C38—C40	−163.6 (3)
C8—C17—C18—C19	−0.6 (3)	C33—C34—C39—C38	−171.9 (2)
C30—C17—C18—C19	−177.5 (2)	C35—C34—C39—C38	7.2 (4)
C8—C17—C18—C28	170.7 (3)	C33—C34—C39—C30	8.9 (4)
C30—C17—C18—C28	−6.3 (5)	C35—C34—C39—C30	−171.9 (2)
C10—C9—C19—C18	175.7 (3)	C37—C38—C39—C34	−9.1 (4)
C8—C9—C19—C18	−2.1 (3)	C40—C38—C39—C34	158.2 (2)
C10—C9—C19—C20	5.2 (6)	C37—C38—C39—C30	170.0 (3)
C8—C9—C19—C20	−172.6 (3)	C40—C38—C39—C30	−22.7 (4)
C17—C18—C19—C9	1.8 (3)	C31—C30—C39—C34	−12.4 (4)
C28—C18—C19—C9	−172.2 (2)	C17—C30—C39—C34	162.6 (2)
C17—C18—C19—C20	175.8 (2)	C31—C30—C39—C38	168.5 (2)
C28—C18—C19—C20	1.8 (3)	C17—C30—C39—C38	−16.5 (4)
C9—C19—C20—C21	−7.3 (6)	C37—C38—C40—O3	125.5 (3)
C18—C19—C20—C21	−177.9 (3)	C39—C38—C40—O3	−42.2 (4)
C9—C19—C20—C29	169.8 (3)	C37—C38—C40—O4	−50.1 (3)
C18—C19—C20—C29	−0.7 (3)	C39—C38—C40—O4	142.2 (3)

### Hydrogen-bond geometry (Å, º)

Cg1 is the centroid of the C18–C20/C28/C29 ring, Cg2 is the centroid of the C24–C29 ring and Cg3 is the centroid of the C11–C16 ring.

**Table:**

*D*—H···*A*	*D*—H	H···*A*	*D*···*A*	*D*—H···*A*
O4—H4′···O3^i^	0.84 (1)	1.81 (1)	2.649 (3)	177 (5)
C26—H26)···O3^ii^	0.93	2.52	3.416 (4)	163
C32—H32···O2^iii^	0.93	2.47	3.301 (4)	149
C35—H35···Cl2^iv^	0.93	2.74	3.619 (3)	157
C2—H2···*Cg*1^v^	0.93	2.87	3.577 (3)	134
C12—H12···*Cg*2^vi^	0.93	2.84	3.725 (3)	160
C21—H21···*Cg*3	0.93	2.57	3.425 (3)	152

Symmetry codes: (i) −*x*, −*y*+1, −*z*+2; (ii) −*x*+1, −*y*+1, −*z*+2; (iii) *x*+1, *y*, *z*; (iv) −*x*+1, −*y*, −*z*+3; (v) −*x*+1, −*y*, −*z*+2; (vi) *x*−1, *y*, *z*.
